# The invasive plant, *Brassica nigra*, degrades local mycorrhizas across a wide geographical landscape

**DOI:** 10.1098/rsos.150300

**Published:** 2015-09-09

**Authors:** Sepideh Pakpour, John Klironomos

**Affiliations:** Department of Biology, University of British Columbia, Okanagan Campus, Kelowna, British Columbia, Canada V1V 1V7

**Keywords:** *Brassica nigra*, mycorrhizal fungi, symbiosis

## Abstract

Disruption of mycorrhizal fungi that form symbioses with local native plants is a strategy used by some invasive exotic plants for competing within their resident communities. Example invasive plants include *Alliaria petiolata* (garlic mustard) and *Brassica nigra* (black mustard), both non-mycorrhizal plants in the Family Brassicaceae. Although there is clear evidence for mycorrhizal degradation, it is not known if such an effect is widespread across the naturalized range. In this study, we tested the ability of black mustard to degrade the local mycorrhizal symbiosis and supress the growth of native flora from across a variety of locations where black mustard has invaded. We found that the effects on mycorrhizal fungi and on the growth of native plants were consistently negative at the various sites. The present results indicate that degradation of the mycorrhizal symbiosis by black mustard is of general significance, and may be highly problematic considering the large range that it has occupied in open fields across North America.

## 1. Introduction

Invasive exotic plants may negatively impact the structure and functioning of communities and ecosystems via a number of mechanisms [1–4]. One mechanism is the degradation of local mycorrhizal fungi that form symbioses with many native plants [5]. Many plants depend on this symbiosis for nutrient acquisition and pathogen protection [6]. It has been suggested that many invasive plants may negatively impact the symbiosis [5,7], as they are often less mycorrhizal-dependent than their native neighbours. Examples of such invaders in North America are *Alliaria petiolata* (garlic mustard) and *Brassica nigra* (black mustard), both non-mycorrhizal plants in the Family Brassicaceae [7–10]. Garlic mustard is abundant in forest edges and closed-canopy forest understory in eastern North America, while the black mustard is more widespread and grows in fields and open areas across all of North America [11]. Studies have demonstrated that these exotic invasive plants may disrupt mycorrhizal associations by producing secondary compounds that directly affect mycorrhizal fungal spore germination, growth and infectivity potential, and hence indirectly impact the growth of native flora that depend on the symbiosis for their growth [7–11]. However, it is not shown if such effects are widespread across the naturalized range. In this study, we have focused on black mustard and conducted a series of comparative field studies at 10 different locations across North America, as well as in the greenhouse. We investigated the effect of black mustard on local arbuscular mycorrhizal (AM) fungi and growth of local native plants across a variety of locations, where black mustard has invaded.

## 2. Material and methods

The objective of study 1 was to determine whether the roots of field-collected black mustard and native plants were colonized by AM fungi. This was done at 10 different locations across the USA. The locations included Kingman, AZ (native plant tested: *Heterotheca subaxillaris* Lam.), San Diego, CA (native plant tested: *Encelia farinosa* A. Gray), Chicago, IL (native plant tested: *Symphyotrichum novae-angliae* L.), Detroit, MI (native plant tested: *Symphyotrichum oolentangiense* Riddell), Socorro, NM (native plant tested: *Aster laevis* L.), Buffalo, NY (native plant tested: *Erigeron strigosus* Muhl), Dayton, OH (native plant tested: *Solidago altissima* L.), Newport, OR (native plant tested: *Symphyotrichum ascendens* Lindl), Richmond, VA (native plant tested: *Chrysopsis mariana* L.) and Seattle, WA (native plant tested: *Achillea millefolium* L.). We found black mustard invasions at each of the 10 sites. For each of the plant species, we sampled when the target species comprised more than 90% of the individuals shoots within a 5×5 m plot. Roots were collected from 10 randomly chosen individuals of each species, separated from the soil, stained with Chlorazol Black E and analysed for per cent mycorrhizal colonization by AM fungi [12]. The remaining soil was then used to measure mycorrhizal infectivity potential using a *Sorghum bicolor* bioassay as described by Klironomos *et al*. [13].

The objective of study 2 was to determine whether a local history of black mustard is associated with reduced mycorrhizal colonization and growth in native plants. Soils were collected from the invaded and uninvaded areas at each location (see study 1 above), brought to the laboratory and screened to remove coarse roots and debris. Two soil treatments were prepared: (i) soil with a history of black mustard, and (ii) soil without a history of black mustard. Eight-inch pots were filled with one of the two soil types. For each soil type, we grew single seedlings of each of the 10 native plants. Each native plant species was replicated 10 times, and thus 100 pots for each soil type. In this study, the experimental unit (replicate) was considered to be the plant species (*n*=10 for each soil type). Any replication within plant species for each soil type was considered to be a subsample, and thus not a true replicate. Pots were randomly placed on a greenhouse bench. After five months of growth, shoots and roots were harvested, dried at 60^°^C for 72 h, and weighed to determine their biomass. An approximately 2 g subsample of roots from each seedling was extracted, stained with Chlorazol Black E and analysed for per cent mycorrhizal colonization by AM fungi [12].

The objective of study 3 was to repeat study 2, but to more explicitly test whether any variation in mycorrhizal colonization and growth in native plants *is caused by* the presence of black mustard. Using eight-inch pots under greenhouse conditions, two treatments (*n*=10) were prepared: (i) soils previously trained with one of the native plants, and (ii) soils previously trained with black mustard. Pre-germinated seedlings of each native species were planted in each of these two treatments. After five months of growth, plants were harvested, biomass was determined and roots were assessed for mycorrhizal colonization as in study 2.

The objective of study 4 was to determine whether black mustard produces antifungal compounds that negatively affect mycorrhizal fungal growth. For this objective, root extracts (soaking the roots in distilled water for 48 h) were prepared from the following plant species: (i) *B. nigra*, (ii) *H. subaxillaris*, (iii) *En. farinosa*, (iv) *Sy. novae-angliae*, (v) *Sy. oolentangiense*, (vi) *As. laevis*, (vii) *Er. strigosus*, (viii) *So. altissima*, (ix) *Sy. ascendens*, (x) *C. mariana* and (xi) *Ac. millefolium*. We tested the effect of the above plant root extracts (11 treatments) on the spore germination of a common AM fungus, *Rhizophagus irregulare* using microcosms in the laboratory as described in Stinson *et al.* [7]. A water control was also included to give 12 treatments.

Student’s *t*-tests (studies 1B, 2 and 3) and analysis of variance (study 4) were conducted in Statistica (StatSoft, 2013).

## 3. Results and discussion

**Figure 1.**
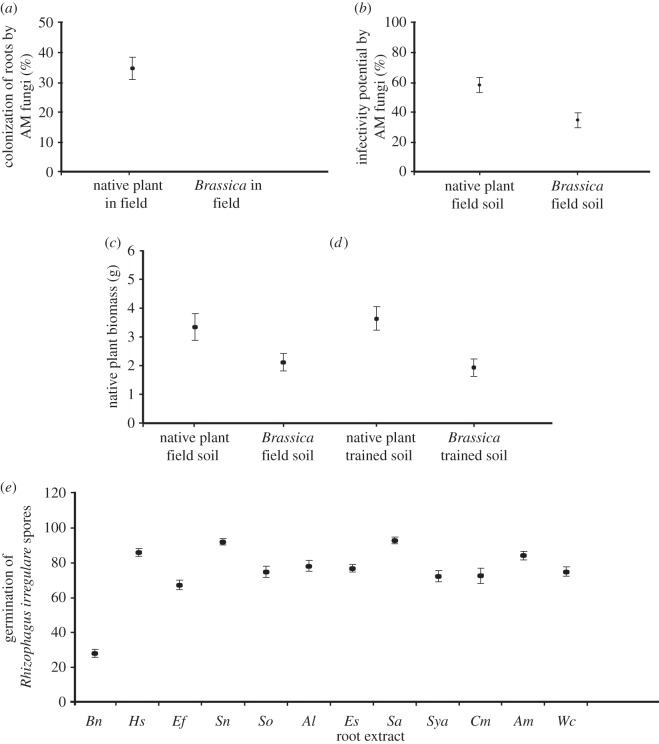
(*a*) Colonization of roots by AM fungi in native plants versus *Brassica nigra* in the field (study 1). (*b*) Infectivity potential by AM fungi in soil underneath native plants versus *B. nigra* (study 1). (*c*) The effect of field soil that previously contained native plants versus *B. nigra* on the growth of native plants (study 2). (*d*) The effect of soil trained (in the greenhouse) by native plants versus *B. nigra* on the growth of native plants (study 3). (*e*) The effect of root extracts on the germination of AM fungal spores. *Bn*, *Brassica nigra*; *Hs*, *Heterotheca subaxillaris*; *Ef*, *Encelia farinosa*; *Sn*, *Symphyotrichum novae-angliae*; *So*, *Symphiotrichum oolentangiense*; *Al*, *Aster laevis*; *Es*, *Erigeron strigosus*; *Sa*, *Solidago altissima*; *Sya*, *Symphyotrichum ascendens*; *Cm*, *Chrysopsis mariana*; *Am*, *Achillea millefolium*. Sample size for each of the above studies is *n*=10 per treatment level. All plots represent the *mean*±*s*.*e*.

Our results clearly demonstrated a consistent negative impact of black mustard on the mycorrhizas of local native plants at the various sites, mainly through phytochemical inhibition. In study 1, we found that all native plant species in the field were colonized by AM fungi. By contrast, we did not detect any colonization of roots by AM fungi in black mustard (figure 1*a*). In addition, the potential for plants to form mycorrhizas was significantly higher (*t*_18_=3.62, *p*=0.001, s.e.m.=4.67 and 4.54) in the native plant field soil than the mustard-invaded field soil (figure 1*b*).

In studies 2 and 3, native plants were significantly less productive when grown in the invaded field soil (*t*_18_=2.48, *p*=0.02, a reduction of 35.5%; figure 1*c*) as well as in the soil that was trained by black mustard under greenhouse conditions (*t*_18_=3.35, *p*=0.003, a reduction of 47.8%; figure 1*d*). Similarly, we observed that native plants displayed significantly lower AM fungal colonization of their roots when planted in the invaded field soil (39.5% decrease, data not shown) as well as the soil trained by black mustard (49.2% decrease, data not shown). Finally, it was previously shown that there is a phytochemical basis to black mustard’s antifungal effects [10,12]. We also found significantly reduced fungal spore germination rates when they were exposed to extracts of black mustard compared to when they were exposed to extracts of any of the native plants (*F*_11,108_=58.44, *p*=0.001; figure 1*e*).

These results show that degradation of the mycorrhizal symbiosis by black mustard is of general significance, and may be highly problematic considering the large range that it has occupied in open fields across North America. This also points to the possibility of an overall strategy by members of the Family Brassicaceae, although clearly we need to study additional species before making any broader conclusions. This Family is one of the 10 most economically important plant Families in North America that are widely used in agriculture as sources of oil (e.g. *Brassica napus*, *Brassica rapa* and *Brassica juncea* as sources of canola and industrial oil), vegetables (e.g. cole-crops (*Brassica oleracea*), swede or rutabaga (*B. napus*), turnip (*B. rapa*), radish (*Raphanus sativus*)), mustard condiments and fodder [14]. However, consequences of these intensive cultivations on the abundance and composition of local AM fungi as well as the growth rate of local mycorrhizal-dependent native plants and biological diversity in native communities is not clear. In addition, such intensive cultivation may exacerbate invasion by members of the Brassicaceae, which consequently may cause an inevitable change on the activity of soil microorganisms, nutrient cycling and plant–insect interactions. There is a need for additional research for more informed agricultural decisions over large spatial scales to avoid potential negative impacts of members of the Brassicaceae on native plant communities.

## Supplementary Material

Supplementary Document-Original Data.

## Data accessibility

Data are available in the electronic supplementary material.
