# Supplementary material for: The invasive plant, Brassica nigra, degrades local mycorrhizas across a wide geographical landscape
Source: R Soc Open Sci. 2015 Sep 9;2(9):150300. doi: 10.1098/rsos.150300 (PMC4593686; doi:10.1098/rsos.150300)
Supplement: Supplementary Document-Original Data. [file rsos150300supp1.docx]

**Supplementary Document**

**Supplementary 1:** Data for the colonization of roots by AM fungi in native plants vs *Brassica nigra* in the field (study 1)

**Supplementary 2:** Data for the Infectivity potential by AM fungi in soil underneath native plants vs *Brassica nigra* (study 1)

**Supplementary 3:** Data for the effect of field soil that previously contained native plants as well as soil trained (in the greenhouse) by native plants vs *Brassica nigra* on the growth of native plants (Study 2 and 3).

**Supplementary 4:** Data for The effect of root extracts on the germination of AM fungal spores.
